# Phylogenetic Distribution and Prevalence of Genes Encoding Class I Integrons and CTX-M-15 Extended-Spectrum *β*-Lactamases in *Escherichia coli* Isolates from Healthy Humans in Chandigarh, India

**DOI:** 10.1371/journal.pone.0112551

**Published:** 2014-11-19

**Authors:** Chetna Dureja, Sakshi Mahajan, Saumya Raychaudhuri

**Affiliations:** Institute of Microbial Technology, Council of Scientific and Industrial Research, Molecular Biology Division, Chandigarh, 160036, India; Wilfrid Laurier University, Canada

## Abstract

*Escherichia coli* is generally considered as a commensal inhabitant of gastrointestinal tract of humans and animals. The aim of this study was to gain insight on the distribution of phylotypes and presence of genes encoding integrons, extended *β*-lactamases and resistance to other antimicrobials in the commensal *E. coli* isolates from healthy adults in Chandigarh, India. PCR and DNA sequencing were used for phylogenetic classification, detections of integrase genes, gene cassettes within the integron and extended *β*-lactamases. The genetic structure of *E. coli* revealed a non-uniform distribution of isolates among the seven phylogenetic groups with significant representation of group A. Integron-encoded integrases were detected in 25 isolates with class 1 integron-encoded *intI*1 integrase being in the majority (22 isolates). The gene cassettes identified were those for trimethoprim, streptomycin, spectinomycin and streptothricin. The *dfrA12*-*orfF-aadA*2 was the most commonly found gene cassette in *intI*1 positive isolates. Phenotypic assay for screening the potential ESBL producers suggested 16 isolates to be ESBL producers. PCR detection using gene-specific primers showed that 15 out of these 16 ESBL-producing *E. coli* harboured the *bla*_CTX-M-15_ gene. Furthermore, molecular studies helped in characterizing the genes responsible for tetracycline, chloramphenicol and sulphonamides resistance. Collectively, our study outlines the intra-species phylogenetic structure and highlights the prevalence of class 1 integron and *bla*_CTX-M-15_ in commensal *E. coli* isolates of healthy adults in Chandigarh, India. Our findings further reinforce the relevance of commensal *E. coli* strains on the growing burden of antimicrobial resistance.

## Introduction

*Escherichia coli*, a commensal of the intestinal tract of vertebrate gut, is one of most well studied model organisms and remains a workhorse in molecular biology. Though a prominent member of gut microbiota, the organism **is** causative agent of life threatening diarrheal diseases, extra-intestinal infections and found to be associated with systemic diseases such as inflammatory bowel syndrome and colorectal cancer [1]–[5]. The composition and genetic structure of *E. coli* is largely dependent on climate conditions, dietary habits and host genetic factors. At present, *E. coli* populations are structured in seven major phylogenetic groups namely A, B1, B2, C, D, E and F. Strains responsible for extra-intestinal infection are far more likely to be member of phylogroups B2 or D than A or B1 [6]. Phylo-groups E and F contains strains, of which O157:H7 is the best known member, and form a sister group to phylo-group B2 respectively. The phylo-group C is closely related but distinct from phylo-group B1 [7].

Much of the studies on antimicrobial resistance and integron associated antimicrobial resistance has focused on pathogenic *E. coli*, even though preponderance of data has clearly documented the frequent presence of genes encoding extended-spectrum-*β-*lactamases and class 1 integrons in commensal *E. coli* strains from healthy human populations of diverse geographic locations [8]–[10]. In addition research has documented the exchange of the genes which confer drug resistance between normal flora and pathogenic *E. coli* and *Salmonella* and in turn the dissemination and the development of multidrug resistance in bacterial population [8], [11], [12].

Until now, data on the distribution of various phylotypes and occurrence of antimicrobial resistance determinants in commensal *E. coli* strains from healthy humans of Indian population are quite scarce; this motivated us to embark on the present study.

## Materials and Methods

### Ethics statement

This study and its procedures were approved and were in accordance with the guidelines of Institutional Bioethics Committee (IBSC), clearance number IBSC/2012-2/19. All the participants were explained the purpose of the study. Participation of all was voluntary and their written consent was obtained. The IBSC committee approved the consent procedure, and the records of the written consent are maintained in our lab.

### Study design, sampling and *E. coli* identification

This study analyzed *E. coli* fecal samples of 102 healthy adults of age 18–35 years (80 males and rest are female) collected during the period of November 2012 to December 2013 from Chandigarh, an urban city in Northern region of India with humid subtropical climate. In the population under study 49 adults were either lacto-ovo-vegetarian or lactovegetarian and 53 were semi vegetarian that is they eat meat once a week. The maximum volunteers were research scholars and remaining was working class. None of the volunteers were suffering from any systemic or diarrheal diseases. They had not been treated with any antibiotics for at least 3 months preceding the collection of fecal sample.

In order to harvest *E. coli* isolates, freshly voided stools were collected from the subjects, placed into sterile sealable plastic containers and transported to the laboratory for processing within 2–4 h of collection. Stool samples were streaked on MacConkey agar (Difco, USA) and incubated aerobically for 18 h at 37°C. One lactose fermenting pink colony per sample was randomly picked. *E. coli* strains were further confirmed by complete 16S rDNA sequencing coupled with standard biochemical test obtained from National Institute of Cholera and Enteric Disease (NICED), Kolkata, India (a WHO accredited laboratory). The phylogenetic group of all 102 isolate was determined according to Clermont *et al*. [7].

### Antimicrobial susceptibility testing and Screening for ESBL

All 102 isolates were tested for the susceptibility using the standard Kirby-Bauer disk diffusion method against 6 classes of antimicrobials at their breakpoint concentration in accordance with the Clinical and Laboratory Standards [13]. The 6 classes of antimicrobials used were aminoglycosides (kanamycin, gentamycin, streptomycin), sulfonamides (sulphafurazole, co-trimoxazole), tetracycline, fluroquinolones (nalidixic acid, ciprofloxacin), chloramphenicol and beta-lactams (ampicillin, cefotaxime, ceftriaxone, ceftazidime, azotreonam, cefepime imipenem meropenem). Isolates that were not susceptible to anyone of the oxyimino-cephalosporins were tested for ESBL production by the double disc synergy test and the combination disk test in accordance with the recommendations of the CLSI. Resistance to antimicrobials belonging to ≥3 classes of antimicrobials was considered as multidrug resistance (MDR). The isolates that showed resistance towards the meropenem was checked for carbapenemase production by modified Hodge test [14]. The antibiotic disks used were purchased from HiMedia Lab. Ltd., India.

### Identification of resistance genes

Isolates that screened positive for ESBL production were subjected to molecular screening for five *β-*lactamase genes namely, *bla*_VEB-1_, *bla*_OXA-10_, *bla*_CTX-M_, *bla*_TEM_ and *bla*_SHV_. DNA was isolated and PCR was performed using specific primer pairs to screen for *β*-lactamase genes [15]. DNA sequencing of resistance genes was performed to validate their identities and further subgrouping. All Sequences were analyzed using the BLAST software (http://www.ncbi.nlm.nih.gov/blast).

All the isolates that showed resistance or moderate susceptibility to tetracycline, chloramphenicol and sulfonamides were checked for the presence of genes encoding resistance to these antimicrobials. Tetracycline resistant samples were checked for presence of *tetA*, *tetB*, *tetC*, *tetD*, *tetE* and *tetG* whereas chloramphenicol resistant strains were examined for the presence of *cat1*, *cat2*, *cat3* and *cmlB* [16]. Sulfonamides resistant samples were tested for *sul1*, *sul2* and *sul3* [16].

### Integron analysis and characterization of inserted gene cassettes

PCR was performed to check the presence of class 1, 2 and 3 integrons in all 102 isolates using primers specific for the integron integrase genes *intI*1, *intI*2 and *intI*3 [17]. Strains that were positive for *intI*1 and/or *intI*2 gene/s were subsequently subjected to PCR for amplification of the variable region of class 1 and class 2 integrons respectively [16]. Amplified products corresponding to the gene cassettes within the integrons were then subjected for nucleotide sequencing and confirmed by BLAST software.

## Results

### Phylogenetic grouping of *E. coli*

Our data reveal the predominance of phylogenetic group A (55 isolates) followed by phylogenetic groups B1 (24 isolates), D (7 isolates), F (7 isolates), B2 (4 isolates), C (3 isolates) and E (1 isolate). One isolate remain unclassified as it was negative for all the genes in quadruplex PCR.

### Prevalence of antimicrobial resistance and distribution of CTX-M extended-spectrum *β-*lactamases in *E. coli* from fecal samples

**Figure 1:**
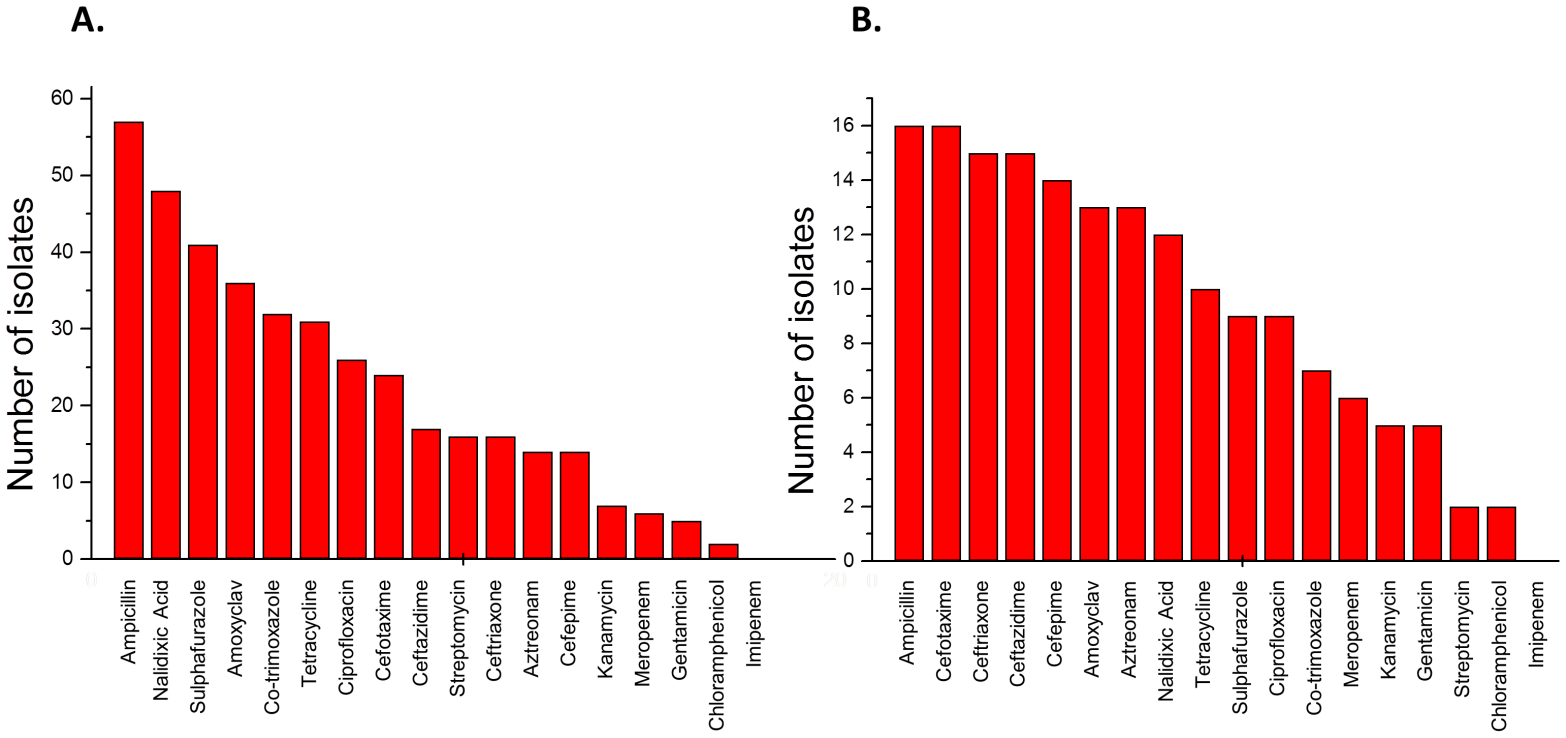
Antimicrobial resistance pattern as interpreted according to the CLSI guidelines (A) of 102 commensal *E. coli* (B) of isolates positive for ESBL phenotype.

Susceptibility test on all 102 isolates against 18 antimicrobial drugs showed the carriage of multidrug resistance in 37 isolates. Resistance was most frequently observed to ampicillin (57 isolates), nalidixic acid (48 isolates), sulphafurazole (41 isolates), amoxyclav (36 isolates), co-trimoxazole (32 isolates) and tetracycline (31 isolates). In contrast, resistance to ciprofloxacin, streptomycin, azotreonam, kanamycin, gentamicin and chloramphenicol were found in 26, 16, 14, 7, 5 and 2 isolates respectively (Figure 1A). It should be highlighted that, resistance to the antimicrobials belonging to the class of fluroquinolones and sulphonamides was much higher than the aminoglycosides and chloramphenicol. 25 isolates were resistant to at least one of the third generation cephalosporins used in the study. 14 isolates were resistant to all of the third and fourth generation cephalosporins tested in this study. Isolates resistant to cefotaxime, ceftriaxone and ceftazidime were 24, 16 and 17 respectively, whereas 14 isolates were resistant to cefepime- a fourth generation cephalosporin. Resistance towards carbapenems was not common, 6 isolates were resistant to meropenem however, none of the isolate was positive in modified Hodge test used to screen carbapenemase. No isolate showed imipenem resistance.

We analyzed the antimicrobial resistance combination pattern in special reference to classes of antimicrobial. The most common resistance pattern were fluroquinolones-beta lactams (33 isolates), sulfonamides-beta lactams (30 isolates) and sulfonamides- fluroquinolones (23 isolates)

Phenotypic assay (double disc synergy test and the combination disk test) for screening the potential ESBL producers suggested 16 isolates to be ESBL producers. We determined that 15 out of these 16 ESBL-producing *E. coli* harbored the *bla*_CTX-M-15_ gene (Table 1). *bla*_TEM-1_ enzyme was also identified in seven of these *bla*_CTX-M-15_ producers. *bla*_OXA-10_, *bla*_SHV-like_ and *bla*_VEB-like_ were not found in any of the isolates. Apart from resistance to beta lactams, ESBL producers showed higher resistance to fluoroquinolone, tetracycline and sulfonamide class of antibiotics (Figure 1B).

**Table 1:** Distribution of isolates positive for ESBL gene, integron and gene cassettes among phylogenetic groups of *E. coli.*

	Phylogenetic group							All groups
	A	B1	B2	C	D	E	F	
**Extended spectrum ** ***β-*** **lactamase**								
* bla*_CTX-M-15_	9	1	-	-	3	-	2	15
**Integrons and Gene cassette array**								
Gene cassette arrays in *intI-1* positive isolates								
* dfrA12*-*orfF-aadA*2	2	3	-	1	-	-	1	7
* dfr17-aadA*5	2	-	-	-	1	-	-	3
* dfrA*1-*aadA*1	-	1	-	-	-	-	-	1
* dfrA*5	2	-	-	-	-	-	-	2
* dfrA*7	1	-	-	-	-	-	-	1
Gene cassette arrays in *intI-2* positive isolates								
* dfrA*1- *sat*1-*aadA*1	1	-	-	1	2	-	-	4

Data are given as numbers of isolates.

### Integron and gene cassettes in *E. coli* isolates: Prevalence of class I integrons

Previous studies have clearly evident the emergence of integron associated antibiotic resistance in intestinal *E. coli* strains. We observed the prevalence of class 1 integron (22 isolates) over class 2 integron (4 isolates) while rarely described class 3 was absent in our commensal *E. coli* isolates. It should be noted that one isolate from our study harbored both class 1 and 2 integron genes. Blast analysis of sequenced products of variable region of class1 and class 2 integrons demonstrated the presence of six different cassette combinations for nine different genes. These genes encode for the resistance to trimethoprim (*dfrA*1, *dfrA*5, *dfrA*7, *dfrA*12 and *dfrA*17), aminoglycosides (*aadA*1, *aadA*2 and *aadA*5) and streptothricin (*satA*1) (Table 1). Out of 22 *intI*1-positive isolates 7 carried *dfrA12*-*orfF-aadA*2 cassettes; three isolate harbored *dfr17-aadA*5 gene cassette; one was found to contain *dfrA1*-*aadA1* cassette, two isolates contained *dfrA*5 and one had *dfrA*7. All 4 *intI*2-positive strains showed the presence of cassette array usually associated with the integron on Tn7: *dfrA*1-*sat*1-*aadA*1. In our isolates, gene cassettes found in the integron conferred resistance to trimethoprim, streptomycin and **streptothricin**. Taken together, our analysis revealed that presence of integron in commensal *E. coli* isolates from healthy subjects that are free of recent antimicrobial exposure.

### Detection of genes encoding resistance to tetracycline, chloramphenicol and sulfonamide

Out of 41 tetracycline resistant or moderate susceptible isolates, 25 isolates were carrying *tetA*. 16 and 1 isolate was PCR positive for *tetB* and *tetD* respectively. One of these isolate was carrying both *tetA* and *tetB*. Both of the chloramphenicol resistant isolates were carrying *cat1* gene. Isolates that were resistant for either or both co-trimoxazole and sulphafurazole were tested for three allelic variation of the *sul* gene. The *sul* gene was found in 36 isolates. The *sul2* was the most common gene present in 26 isolates, whereas *sul1* was found in 18 isolates. 8 of these isolates were carrying both *sul1* and *sul2* genes. No isolate was tested positive for the presence of *sul3*. Further classification of *sul* allele showed that 20 out of 25 integrase 1 and/or 2 positive isolates carried atleast one *sul* gene. 16 out of 36 *sul* positive isolates were not tested positive for the presence of any *intI* genes.

## Discussion

In this work, we were driven by a desire to understand the phylogenetic architecture and antimicrobial resistance pattern of commensal *E. coli* from healthy population in Chandigarh, India. In this connection, our data are in congruence with previous reports and clearly witnessed the predominance of strains belonging to phylotype A as well as prevalence of class 1 integron and CTX-M-15 ESBL positive strains in healthy Indian population [18]–[21].

It should be highlighted that our geographically distinct population is significantly different in terms of percentage of isolates in each phylogenetic groups [18]–[20]. We observed the prevalence of *E. coli* strains belonging to phylogeny A and B1 over other phylotype reflecting similar pattern as seen in case of other population [18], [22], [23]. Interestingly, we found more D over B2 whereas earlier study describes more B2 phylotype than D [7], [20]. This alteration could be explained in terms of differences in dietary habits, environmental conditions or host genetic factors. Our data further support the earlier reports where it was concluded that in tropical climate phylogroup A dominate over B1 and prevalence of B2 and D is very low, suggesting the role of environmental factors in defining the genetic structure of *E. coli* in a given population [19].

The prevalence of class I integron in our isolates is in agreement with previous report where frequency of class 1 integron is reported much more than class 2 [21]. In the class 1 integron-positive isolates we found eight different gene cassettes in five different combinations, namely *dfrA12-orfF-aadA2, dfrA1*-*aadA1, dfrA17-aadA5, dfrA*5 and *dfrA*7. The presence of more than one gene cassettes in 11 of the 22 class 1 integrons positive samples support the literature suggesting that since 1990 there is a prevalence of class 1 integrons carrying multiple gene cassettes [24], [25]. In 8 of the class 1 intergron positive isolates we could not amplify cassette region, which may be due to lack of the 3′-CS. Unlike reports from Taiwan, Tunisia and Madagascar where *dfr17-aad5* combination is most frequently detected, most common cassette found in our isolates is *dfrA12*-*orfF-aadA*2 [26]–[28]. This observation warrants further extensive follow up evaluation in order to determine why some gene cassette combinations are more prevalent than others and their percentage distribution is different around the globe.

Interestingly, CTX-M-15 enzyme was first detected in enterobacterial isolates from patient hospitalized in New Delhi, India [29]. Since then, increasing occurrence of this particular type of ESBL has been reported in clinical *E. coli* isolates from different parts of the world [30]. The prevalence of CTX-M-15 producers in this study is strikingly high. As CTX-M-15 is known to have peculiar association with community-onset *E. coli* infections [31] therefore it may be concluded that CTX-M-15-producing *E*. *coli* have already been established in our area. Phylogenetic B group and serotype O25:H4 has been particularly associated with CTX-M-15, but, in this study 9 of the 15 CTX-M-15 were from phylogroup A and only isolate was of phylogenetic group B. It should be noted that one isolate of phenotypically confirmed ESBL do not have any ESBL encoding gene, this isolate probably produce other ESBL enzyme that were not checked during this study. In addition like earlier studies, we observed high prevalence of fluoroquinolones resistance among ESBL-producing isolates [32], [33]. Many reasons have been suggested in literature for this association but the most possible explanation is the presence of genes of the two resistance mechanisms on the same plasmid. We also observed that all six meropenem resistant isolates are carbapenemase negative but ESBL producers. This acquired resistance could be attributed to many factors including the loosening of outer membrane porins in ESBL positive isolates resulting in reduced carbapenem uptake [34].

Acquisition of tetracycline, sulphonamide and chloramphenicol genes are also of concern. In contrast to earlier reports where *tetB* is reported as the most common gene found [35], [36], our population observed the prevalence of *tetA.* One isolate tested positive for both *tetA* and *tetB.* The difference in the distribution pattern of *tet* gene suggest ecosystem-specific reservoir for resistance gene. Our isolates showed the prevalence of *sul2* gene over *sul1*, the observed pattern of gene frequency distribution (*sul2>sul1>sul3*) corroborate previous studies [37]. Though literature has witnessed the *sul1* gene as consistent marker for the presence of class 1 integron, still we found 11 isolates that were carrying the *intI1* but not the *sul1* gene [38]. Similar finding has been reported earlier where it has been suggested that it is either because of the loss of *sul1* gene region from class 1 integrons or that this gene is carried on another genetic context in the isolates [39]. 14 isolates that were tested resistant or moderately susceptible against the sulphonamides did not harbor any *sul* gene pointing to the probability of other *sul* alleles in these isolates or other resistance mechanism.

There are reports documenting ESBL positive *E. coli* isolates primarily from clinical settings in India [40]–[42]. In this regard, our study remains first report to reveal the status of commensal *E. coli* strains from healthy Indian population and supports the emerging theme of commensal flora mediated antibiotic resistance burden. The simple, low-cost survey of resistance in commensal bacteria like this predicts that antimicrobial resistance and resistance markers are circulating widely in the community and may reflect resistance in circulating pathogens [43]. The data not only underscore the importance of continued surveillance of antibiotic resistance but will also provide information crucial in developing locally appropriate guidelines for efficacious treatment of *E. coli* and other bacterial infections in developing countries like India where there are severely limited therapeutic options. Lastly, the literature is replete with examples how genetic structure as well as antimicrobial carriage of commensal *E. coli* strains is governed by various environmental and host factors. In the light of the present knowledge, future studies will be directed to evaluate the status of *E. coli* strains from diverse geographical and socio-economic Indian population.

## References

[1] Tenaillon O, Skurnik D, Picard B, Denamur E (2010) The population genetics of commensal Escherichia coli. Nat Rev Microbiol 8: 207–217. 20157339 10.1038/nrmicro2298

[2] Rolhion N, Darfeuille-Michaud A (2007) Adherent-invasive Escherichia coli in inflammatory bowel disease. Inflamm Bowel Dis 13: 1277–1283. 17476674 10.1002/ibd.20176

[3] Kaper JB, Nataro JP, Mobley HL (2004) Pathogenic Escherichia coli. Nat Rev Microbiol 2: 123–140. 15040260 10.1038/nrmicro818

[4] Arthur JC, Perez-Chanona E, Muhlbauer M, Tomkovich S, Uronis JM, et al (2012) Intestinal inflammation targets cancer-inducing activity of the microbiota. Science 338: 120–123. 22903521 10.1126/science.1224820 PMC3645302

[5] Cuevas-Ramos G, Petit CR, Marcq I, Boury M, Oswald E, et al (2010) Escherichia coli induces DNA damage in vivo and triggers genomic instability in mammalian cells. Proc Natl Acad Sci U S A 107: 11537–11542. 20534522 10.1073/pnas.1001261107 PMC2895108

[6] Johnson JR, Stell AL (2000) Extended virulence genotypes of Escherichia coli strains from patients with urosepsis in relation to phylogeny and host compromise. J Infect Dis 181: 261–272. 10608775 10.1086/315217

[7] Clermont O, Christenson JK, Denamur E, Gordon DM (2013) The Clermont Escherichia coli phylo-typing method revisited: improvement of specificity and detection of new phylo-groups. Environ Microbiol Rep 5: 58–65. 23757131 10.1111/1758-2229.12019

[8] Skurnik D, Le Menac'h A, Zurakowski D, Mazel D, Courvalin P, et al (2005) Integron-associated antibiotic resistance and phylogenetic grouping of Escherichia coli isolates from healthy subjects free of recent antibiotic exposure. Antimicrob Agents Chemother 49: 3062–3065. 15980401 10.1128/AAC.49.7.3062-3065.2005 PMC1168629

[9] Pallecchi L, Bartoloni A, Fiorelli C, Mantella A, Di Maggio T, et al (2007) Rapid dissemination and diversity of CTX-M extended-spectrum beta-lactamase genes in commensal Escherichia coli isolates from healthy children from low-resource settings in Latin America. Antimicrob Agents Chemother 51: 2720–2725. 17548490 10.1128/AAC.00026-07 PMC1932529

[10] Bailey JK, Pinyon JL, Anantham S, Hall RM (2010) Commensal Escherichia coli of healthy humans: a reservoir for antibiotic-resistance determinants. J Med Microbiol 59: 1331–1339. 20671087 10.1099/jmm.0.022475-0

[11] Leverstein-van Hall MA, Box AT, Blok HE, Paauw A, Fluit AC, et al (2002) Evidence of extensive interspecies transfer of integron-mediated antimicrobial resistance genes among multidrug-resistant Enterobacteriaceae in a clinical setting. J Infect Dis 186: 49–56. 12089661 10.1086/341078

[12] Blake DP, Hillman K, Fenlon DR, Low JC (2003) Transfer of antibiotic resistance between commensal and pathogenic members of the Enterobacteriaceae under ileal conditions. J Appl Microbiol 95: 428–436. 12911689 10.1046/j.1365-2672.2003.01988.x

[13] Clinical Laboratory Standard Institute (2012) Performance Standards for Antimicrobial Susceptibility Testing; Twenty-Second Informational Supplement. Vol. 32. Clinical Laboratory Standard Institute; Wayne, Pennsylvania, USA: 2012.

[14] Ben Nasr A, Decre D, Compain F, Genel N, Barguellil F, et al (2013) Emergence of NDM-1 in association with OXA-48 in Klebsiella pneumoniae from Tunisia. Antimicrob Agents Chemother 57: 4089–4090. 23752514 10.1128/AAC.00536-13 PMC3719753

[15] Cao V, Lambert T, Nhu DQ, Loan HK, Hoang NK, et al (2002) Distribution of extended-spectrum beta-lactamases in clinical isolates of Enterobacteriaceae in Vietnam. Antimicrob Agents Chemother 46: 3739–3743. 12435670 10.1128/AAC.46.12.3739-3743.2002 PMC132739

[16] Chang CY, Lu PL, Lin CC, Lee TM, Tsai MY, et al (2011) Integron types, gene cassettes, antimicrobial resistance genes and plasmids of Shigella sonnei isolates from outbreaks and sporadic cases in Taiwan. J Med Microbiol 60: 197–204. 20947666 10.1099/jmm.0.022517-0

[17] Mazel D, Dychinco B, Webb VA, Davies J (2000) Antibiotic resistance in the ECOR collection: integrons and identification of a novel aad gene. Antimicrob Agents Chemother 44: 1568–1574. 10817710 10.1128/aac.44.6.1568-1574.2000 PMC89914

[18] Duriez P, Clermont O, Bonacorsi S, Bingen E, Chaventre A, et al (2001) Commensal Escherichia coli isolates are phylogenetically distributed among geographically distinct human populations. Microbiology 147: 1671–1676. 11390698 10.1099/00221287-147-6-1671

[19] Escobar-Paramo P, Grenet K, Le Menac'h A, Rode L, Salgado E, et al (2004) Large-scale population structure of human commensal Escherichia coli isolates. Appl Environ Microbiol 70: 5698–5700. 15345464 10.1128/AEM.70.9.5698-5700.2004 PMC520916

[20] Bailey JK, Pinyon JL, Anantham S, Hall RM (2010) Distribution of human commensal Escherichia coli phylogenetic groups. J Clin Microbiol 48: 3455–3456. 20610687 10.1128/JCM.00760-10 PMC2937668

[21] van Essen-Zandbergen A, Smith H, Veldman K, Mevius D (2007) Occurrence and characteristics of class 1, 2 and 3 integrons in Escherichia coli, Salmonella and Campylobacter spp. in the Netherlands. J Antimicrob Chemother 59: 746–750. 17307772 10.1093/jac/dkl549

[22] Ciccozzi M, Giufr M, Accogli M, Lo Presti A, Graziani C, et al (2013) Phylogenetic analysis of multidrug-resistant Escherichia coli clones isolated from humans and poultry. New Microbiol 36: 385–394. 24177300

[23] Li B, Sun JY, Han LZ, Huang XH, Fu Q, et al (2010) Phylogenetic groups and pathogenicity island markers in fecal Escherichia coli isolates from asymptomatic humans in China. Appl Environ Microbiol 76: 6698–6700. 20709835 10.1128/AEM.00707-10 PMC2950456

[24] Yu HS, Lee JC, Kang HY, Ro DW, Chung JY, et al (2003) Changes in gene cassettes of class 1 integrons among Escherichia coli isolates from urine specimens collected in Korea during the last two decades. J Clin Microbiol 41: 5429–5433. 14662921 10.1128/JCM.41.12.5429-5433.2003 PMC309026

[25] Kang HY, Jeong YS, Oh JY, Tae SH, Choi CH, et al (2005) Characterization of antimicrobial resistance and class 1 integrons found in Escherichia coli isolates from humans and animals in Korea. J Antimicrob Chemother 55: 639–644. 15761064 10.1093/jac/dki076

[26] Rakotonirina HC, Garin B, Randrianirina F, Richard V, Talarmin A, et al (2013) Molecular characterization of multidrug-resistant extended-spectrum beta-lactamase-producing Enterobacteriaceae isolated in Antananarivo, Madagascar. BMC Microbiol 13: 85. 23594374 10.1186/1471-2180-13-85 PMC3639105

[27] Chang LL, Chen HF, Chang CY, Lee TM, Wu WJ (2004) Contribution of integrons, and SmeABC and SmeDEF efflux pumps to multidrug resistance in clinical isolates of Stenotrophomonas maltophilia. J Antimicrob Chemother 53: 518–521. 14749340 10.1093/jac/dkh094

[28] Jouini A, Ben Slama K, Vinue L, Ruiz E, Saenz Y, et al (2010) Detection of unrelated Escherichia coli strains harboring genes of CTX-M-15, OXA-1, and AAC(6′)-Ib-cr enzymes in a Tunisian hospital and characterization of their integrons and virulence factors. J Chemother 22: 318–323. 21123154 10.1179/joc.2010.22.5.318

[29] Karim A, Poirel L, Nagarajan S, Nordmann P (2001) Plasmid-mediated extended-spectrum beta-lactamase (CTX-M-3 like) from India and gene association with insertion sequence ISEcp1. FEMS Microbiol Lett 201: 237–241. 11470367 10.1111/j.1574-6968.2001.tb10762.x

[30] Pitout JD, Church DL, Gregson DB, Chow BL, McCracken M, et al (2007) Molecular epidemiology of CTX-M-producing Escherichia coli in the Calgary Health Region: emergence of CTX-M-15-producing isolates. Antimicrob Agents Chemother 51: 1281–1286. 17283198 10.1128/AAC.01377-06 PMC1855502

[31] Muzaheed, Doi Y, Adams-Haduch JM, Endimiani A, Sidjabat HE, et al (2008) High prevalence of CTX-M-15-producing Klebsiella pneumoniae among inpatients and outpatients with urinary tract infection in Southern India. J Antimicrob Chemother 61: 1393–1394. 18356153 10.1093/jac/dkn109 PMC2736628

[32] Katsandri A, Avlamis A, Vasilakopoulou A, Mela V, Kosmidis C, et al (2008) Risk factors for coexistence of fluoroquinolone resistance and ESBL production among Enterobacteriaceae in a Greek university hospital. J Chemother 20: 452–457. 18676225 10.1179/joc.2008.20.4.452

[33] Lautenbach E, Strom BL, Bilker WB, Patel JB, Edelstein PH, et al (2001) Epidemiological investigation of fluoroquinolone resistance in infections due to extended-spectrum beta-lactamase-producing Escherichia coli and Klebsiella pneumoniae. Clin Infect Dis 33: 1288–1294. 11565067 10.1086/322667

[34] Doumith M, Ellington MJ, Livermore DM, Woodford N (2009) Molecular mechanisms disrupting porin expression in ertapenem-resistant Klebsiella and Enterobacter spp. clinical isolates from the UK. J Antimicrob Chemother 63: 659–667. 19233898 10.1093/jac/dkp029

[35] Bryan A, Shapir N, Sadowsky MJ (2004) Frequency and distribution of tetracycline resistance genes in genetically diverse, nonselected, and nonclinical Escherichia coli strains isolated from diverse human and animal sources. Appl Environ Microbiol 70: 2503–2507. 15066850 10.1128/AEM.70.4.2503-2507.2004 PMC383146

[36] Wilkerson C, Samadpour M, van Kirk N, Roberts MC (2004) Antibiotic resistance and distribution of tetracycline resistance genes in Escherichia coli O157:H7 isolates from humans and bovines. Antimicrob Agents Chemother 48: 1066–1067. 14982813 10.1128/AAC.48.3.1066-1067.2004 PMC353164

[37] Blahna MT, Zalewski CA, Reuer J, Kahlmeter G, Foxman B, et al (2006) The role of horizontal gene transfer in the spread of trimethoprim-sulfamethoxazole resistance among uropathogenic Escherichia coli in Europe and Canada. J Antimicrob Chemother 57: 666–672. 16464890 10.1093/jac/dkl020

[38] Antunes P, Machado J, Peixe L (2007) Dissemination of sul3-containing elements linked to class 1 integrons with an unusual 3′ conserved sequence region among Salmonella isolates. Antimicrob Agents Chemother 51: 1545–1548. 17283193 10.1128/AAC.01275-06 PMC1855504

[39] Gundogdu A, Long YB, Vollmerhausen TL, Katouli M (2011) Antimicrobial resistance and distribution of sul genes and integron-associated intI genes among uropathogenic Escherichia coli in Queensland, Australia. J Med Microbiol 60: 1633–1642. 21737542 10.1099/jmm.0.034140-0

[40] Sridhar Rao PN, Basavarajappa KG, Krishna GL (2008) Detection of extended spectrum beta-lactamase from clinical isolates in Davangere. Indian J Pathol Microbiol 51: 497–499. 19008574 10.4103/0377-4929.43739

[41] Jemima SA, Verghese S (2008) Multiplex PCR for bla(CTX-M) & bla(SHV) in the extended spectrum beta lactamase (ESBL) producing gram-negative isolates. Indian J Med Res 128: 313–317. 19052344

[42] Raghunath D (2008) Emerging antibiotic resistance in bacteria with special reference to India. J Biosci 33: 593–603. 19208984 10.1007/s12038-008-0077-9

[43] Seidman JC, Anitha KP, Kanungo R, Bourgeois AL, Coles CL (2009) Risk factors for antibiotic-resistant E. coli in children in a rural area. Epidemiol Infect 137: 879–888. 19000341 10.1017/S0950268808001519 PMC2841309

